# Leveraging innate immune signals in CD8+ T cells to boost antitumor immunity

**DOI:** 10.3389/fimmu.2025.1617773

**Published:** 2025-08-29

**Authors:** Gabriella K. Albert, Phoebe Cao, Eduardo Davila

**Affiliations:** ^1^ Department of Medicine, Division of Medical Oncology, University of Colorado School of Medicine, Aurora, CO, United States; ^2^ University of Colorado Comprehensive Cancer Center, Aurora, CO, United States

**Keywords:** pattern recognition receptors, innate receptor signaling, innate-like stimulation, T cell immunotherapy, antitumor immunity

## Abstract

Pattern recognition receptors (PRRs), traditionally characterized in innate immune cells, are emerging as critical modulators of T cell function. Toll-like receptors (TLRs), STING, RIG-I-like receptors (RLRs), and natural killer receptors (NKRs) are expressed by CD8+ T cells, where they influence various cellular responses. Primarily serving as noncanonical costimulatory signals, TLRs can modulate T cell activation, differentiation, metabolic fitness, and memory formation. RLRs and STING can promote T cell expansion and cytokine production. Both activating and inhibitory NKRs can also alter T cell cytotoxicity and differentiation. As demonstrated in recent advancements, the capacity of these signaling cascades to enhance T cell responses offers promising therapeutic opportunities in cancer. Clinical strategies are being developed to selectively harness each of these pathways, such as TLR and STING agonists to bolster antitumor responses, and NKR-based approaches to amplify cytotoxic function. Additionally, adoptive T cell therapies, such as chimeric antigen receptor (CAR)-T cells, are incorporating these innate signaling components to overcome tumor-mediated immunosuppression, enhance functional longevity, and improve therapeutic efficacy. This review discusses the progress made to characterize the role of T cell intrinsic PRR activity in shaping T cell functions and highlights recent advancements in that leverage innate receptor signaling to enhance the efficacy of cancer immunotherapies.

## Introduction

The innate immune system is an evolutionarily conserved defense that detects pathogenic threats and cellular stress. Central to this system are pattern recognition receptors (PRRs), found on innate immune cells like macrophages and dendritic cells. These receptors recognize pathogen- and damage-associated molecular patterns (PAMPs/DAMPs), triggering inflammatory and type-I interferon responses. Key PRR classes include Toll-like receptors (TLRs), STING, and RIG-I-like receptors (RLRs) (1–3). Natural Killer (NK) cells also express germline-encoded NK receptors (NKRs) that detect stress signals and self-antigens (4). Activation of these receptors initiates innate defense and shapes adaptive immunity.

Initially studied in infections, PRRs also play a vital role in cancer immune surveillance. TLRs and nucleic acid sensors detect fragments of dying cancer cells, promoting immune-mediated tumor clearance (5–7). NKRs enhance antitumor immunity through NK cell cytotoxicity. However, tumors often evade innate immune responses, enabling growth and metastasis. To counter this, PRR agonists have been developed as cancer immunotherapies, also benefiting adaptive immune cells like T cells.

Unlike traditional innate-like T cells (i.e. MAIT, iNKT, γδ T cells), which typically express a limited TCR repertoire, a growing body of literature show that some αβ T cells co-express PRRs such as TLRs, RLRs, STING, and NKRs alongside conventional TCRs, allowing for rapid responses (2, 3, 8). Tumor-specific T cells also express these receptors, with evidence showing innate signaling influences T cell function, differentiation, and survival. Despite this, challenges remain in targeting PRRs specifically in T cells. Cellular engineering approaches to harness T cell-intrinsic innate signaling are emerging to boost antitumor immunity.

While T-cell therapies have transformed cancer treatment, their efficacy is often limited by immunosuppressive tumor environments. Enhancing T cell function via innate receptor signaling offers a complementary strategy. This review explores how PRR pathways activated in T cells influence their fate and antitumor activity, focusing on T cell-intrinsic TLR, STING, RLR, and NKR signaling. We also examine how these pathways intersect with conventional αβ TCR signaling to support CD8+ T cell immunity.

## Toll-like receptors

**Figure 1:**
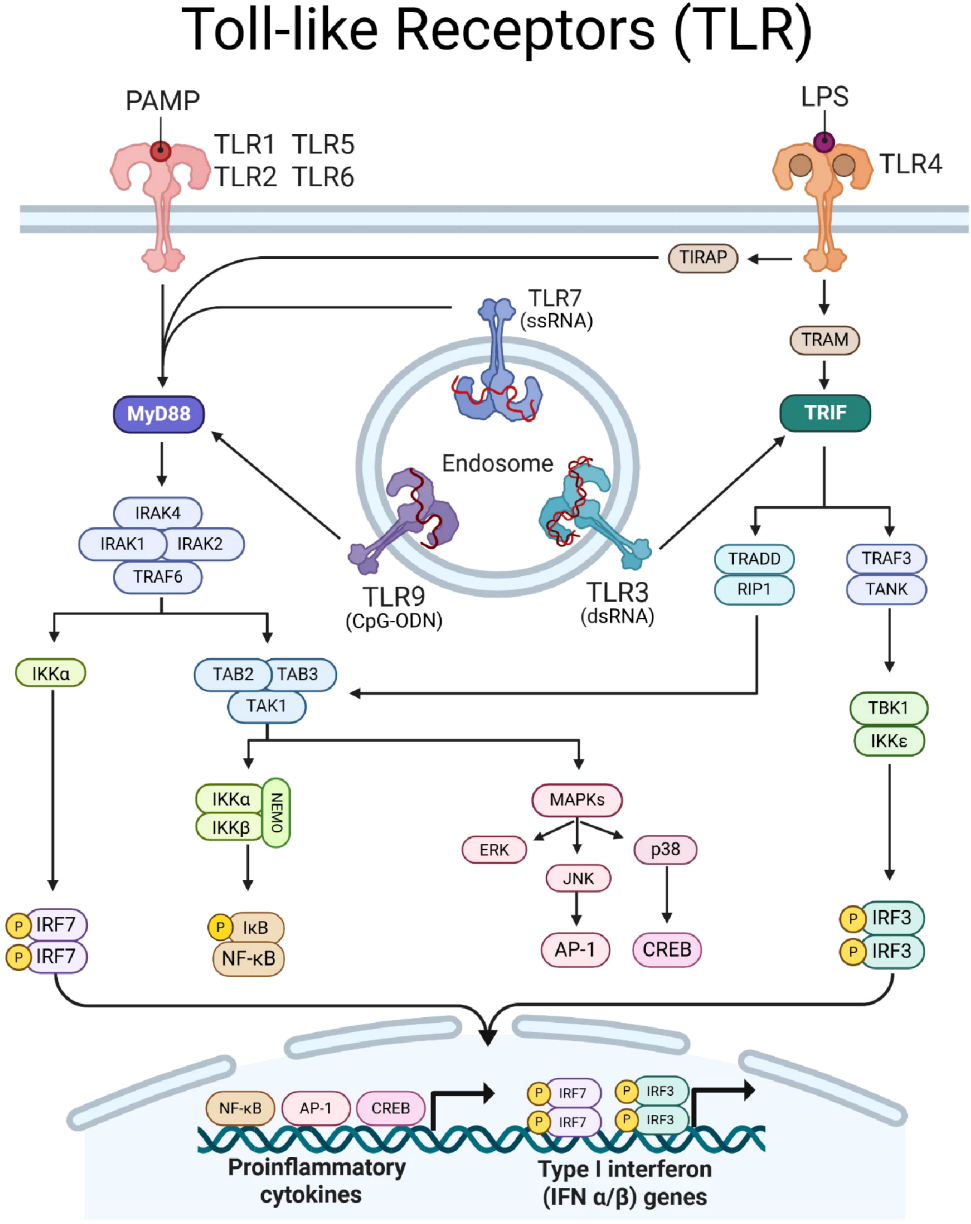
Toll-like receptor (TLR) signaling. TLRs dimerize upon ligation of PAMPs/DAMPs and transduce signal via intracellular adaptor proteins MyD88 (indigo) and/or TRIF (teal). TLR-1, -2, -4, -5, -6, -7, and -9 signal through MyD88. MyD88 forms a Myddosome complex with IRAK4, recruiting IRAK1, IRAK2, and TRAF6. Myddosome formation leads to (1) IKKα activation to induce phosphorylation of IRF7 and (2) recruits TAB2/3 proteins to form a scaffold for TAK1. TAK1 can induce (1) MAPK signaling, leading to ERK and JNK to induce AP-1 and p38 to activate CREB, and (2) can induce the NEMO complex to facilitate phosphorylation of IkB to activate NF-kB. TLR-3 and -4 signal through TRIF, which transduces signal to TRAF3 and TRADD. TRAF3 complexes with TANK to induce TBK1 activity, resulting in phosphorylation of IRF3. TRADD recruits RIP1 to converge with MyD88-depdendent signaling pathways by activating TAK1. Together, TLR signaling results in the translocation of transcription factors IRF7, AP-1, CREB, NF-kB, and IRF3, into the nucleus to drive expression of proinflammatory cytokine and type-I IFN genes. Created with BioRender.com.

TLRs belong to the interleukin-1 receptor (IL-1R)/TLR superfamily and share structural similarities, including an extracellular leucine-rich repeat (LRR) domain responsible for ligand recognition, a transmembrane domain, and a cytoplasmic Toll/IL-1 receptor (TIR) domain essential for mediating downstream signaling (3). There are ten TLRs (TLR1-TLR10) in humans and twelve in mice (TLR1-TLR9, TLR11-TLR13) (3). These receptors are broadly classified based on their cellular localization and ligand specificity (**Figure 1**). Plasma membrane-bound TLRs primarily detect extracellular microbial components such as bacterial lipoproteins, lipopolysaccharides (LPS), and flagellin. TLRs localized to endosomal compartments are mainly involved in recognizing nucleic acids from viruses and bacteria (9).

Upon ligand binding, TLRs will dimerize and recruit adaptor molecules to initiate signaling. TLRs signal through two main adaptor molecules: the myeloid differentiation primary response protein-88 (MyD88) or the TIR domain containing adapter inducing interferon β (TRIF), leading to distinct immune responses (**Figure 1**). The MyD88-dependent pathway is used by all TLRs except TLR3 and recruit’s IL-1 receptor-associated kinases (IRAKs), leading to the activation of TNF receptor-associated factor 6 (TRAF6). This cascade results in the activation of transcription factor (TF) nuclear factor-kappa B (NF-κB) and initiation of MAPK signaling to induce TF activator protein-1 (AP-1). Together, these TFs promote the transcription of inflammatory cytokines such as IL-6, TNF-α, and IL-12. Conversely, the TRIF-dependent pathway is used by TLR3 and TLR4, and leads to activation of interferon response factor, IRF3. MyD88 through TLR7–9 stimulation promotes activation of IRF7 which can cooperate with TRIF-induced IRF3 to promote the production of type I interferons (10).

### TLR agonists amplifies TCR-mediated CD8^+^ T cell function and fitness

In CD8+ T cells, TLR engagement can amplify TCR signaling by enhancing proliferation, survival, cytokine production, and metabolic fitness. Several studies have demonstrated that Pam3CSK4, a TLR1/2 agonist, acts as a potent stimulatory signal in TCR-activated CD8+ T cells, leading to enhanced activation, expansion, cytokine production, and improved antiviral and antitumor responses. Joseph et al. (11) demonstrated that simultaneous TCR and TLR1/2 stimulation via anti-CD3ϵ and Pam3CSK4, respectively, resulted in a 41BB-dependent enhancement of CD8+ T cell expansion and antitumor activity (11). Specifically, Pam3CSK4 was able to drive expression of noncanonical costimulatory surface molecules such as 41BB, OX40, GITR, and LTA (lymphotoxin-alpha) in CD8+ T cells (11). Mechanistically, TLR1/2 activation promoted enhanced NF-κB (p65) and AP-1 (c-Jun) binding to the 41BB promoter, driving its transcription and surface expression (11). TLR1/2 mediated upregulation of 41BB was shown to be dependent on MyD88 and IRAK4 activation as demonstrated in respective knockout murine models (11). Adoptive transfer of TLR1/2 and 41BB agonist (3H3) treated pmel transgenic T cells into B16-F1 melanoma bearing mice resulted in improved tumor rejection and survival (11), highlighting that TLR signaling within CD8+ T cells can boost antitumor immunity.

Mechanistically, Zhang et al. (12) then showed that TLR1/2 signaling operates through a MyD88-dependent pathway, as MyD88-deficient CD8+ T cells failed to respond to Pam3CSK4 stimulation (12). Interestingly, transcriptomic analysis revealed that TLR1/2 activation upregulated IRF4, BATF, and T-bet, which drive metabolic reprogramming towards energetic processes favoring effector activity including glycolysis and glutaminolysis (12). Key glycolytic proteins, glucose transporter 1 (GLUT1), hexokinase 2 (HK2), and lactate dehydrogenase A (LDHa), were also upregulated upon TLR1/2 stimulation (12). Functional assays confirmed that TLR1/2 stimulation enhanced glucose uptake and glutamine metabolism (CD98, Gls2), leading to elevated mitochondrial respiration and ATP production (12). This metabolic shift is essential for enhanced CD8+ T cell activation, as pharmacological inhibition of glycolysis and glutaminolysis suppressed TLR1/2-mediated cytokine production (IFN-γ, IL-2) and proliferation. In a woodchuck hepatitis virus (WHV) model, *in-vivo* Pam3CSK4 administration promoted antigen-specific CD8+ T cell expansion, IFN-γ production, and improved viral clearance, establishing TLR1/2 as a critical metabolic and immunological regulator of CD8+ T cell responses (12).

Similarly, TLR7 has been shown to promote cellular glycolysis, thereby enhancing CD8+ T cell effector function. Li et al. (13) found that stimulation of TLR7 in the presence of TCR signaling resulted in MyD88-dependent activation of the AKT-mTOR pathway, inducing increased expression of glycolytic enzymes, GLUT1, HK2, and LDHa (13). Functional assays confirmed that TLR7-stimulated CD8+ T cells exhibited higher IFN-γ, TNF-α, and IL-2 production, and this response was abolished upon inhibition of glycolysis or mTOR (13). Here, IRF4 was identified the link between TLR7-driven metabolic reprogramming and enhanced effector function, as IRF4-deficient CD8+ T cells failed to upregulate glycolysis and cytokine production following TLR7 stimulation (13). Together, these studies suggest that targeting TLR1/2 and/or TLR7 signaling in CD8+ T cells may enhance their fitness and function through modulating metabolic activity.

In 2020, Imanishi et al. found that effector but not naive CD8+ T cells, responded to Pam3CSK4, and TLR2/6 agonist, Fibroblast stimulating lipopeptide-1 (FSL-1) (14). This specificity to activated T cells was due to the expression of Toll/IL-1 receptor domain-containing adaptor protein (TIRAP), which is induced by TCR and IL-2 mediated mTORC1 signaling (14). TIRAP was required for Pam3CSK4 and FSL-1-mediated activation of NF-κB and ERK pathways, leading to enhanced IFN-γ production (14). Interestingly, Salerno et al. (15) showed that the increased IFN-γ production in CD8+ T cells was specific to TLR1/2 heterodimerization (Pam3CSK4) rather than TLR2/6 (zymosan) (15). Indeed, treatment with Pam3CSK4 lowered the antigen threshold required for effector cytokine production, while zymosan treatment yielded no difference (15). Mechanistically, TLR1/2 activation supported both *de novo* transcription and mRNA stability of cytokine genes, synergizing with TCR engagement to prolong IFN-γ mRNA half-life and enhance CD8+ T cell polyfunctionality (15). The study also revealed that TLR1/2-mediated co-stimulation enhanced antitumor CD8+ T cell function when encountering tumor cells (15), further supporting the potential for leveraging TLR1/2 signaling in immunotherapeutic strategies.

### MyD88 and IRAK-4 are critical adapters in TLR and TCR signaling

Independent of ligand binding, TLR signaling adaptor proteins also play crucial roles in modulating CD8+ T cell differentiation and function. Key mediators such as IRAK-4 and MyD88 integrate innate immune signaling with TCR-driven responses, influencing cytokine production, survival, and effector differentiation. McDonald et al. (16) established the importance of IRAK-4 in linking TCR signaling to NF-κB and MAPK pathways in CD8+ T cells. In CD8+ T cells from IRAK-4-deficient patients showed impaired NF-κB and p38 MAPK activation, resulting in failure of TCR stimulation to upregulate activation markers CD25 and CD69 (16). Functionally, IFN-γ production was significantly reduced, while IL-2 secretion remained unaffected, suggesting that IRAK-4 selectively regulates inflammatory cytokine responses (16). However, phorbol 12-myristate 13-acetate (PMA) and ionomycin stimulation rescued NF-κB and MAPK activation, confirming that the defect was in proximal TCR signaling rather than downstream cascades (16). These findings establish IRAK-4 as a critical adapter linking TCR engagement to CD8+ T cell activation and inflammatory cytokine production. Similarly, independent of TLR engagement, MyD88 plays an essential role in CD8+ T cell survival and effector activity. Matsuoka et al. (17) demonstrated that MyD88 signaling is necessary for effector T cell differentiation as MyD88-deficient CD8+ T cells exhibited impaired IFN-γ signaling (17). Moreover, using an allogenic bone marrow transplantation model, the study found that while proliferation was unaffected by MyD88-deficient CD8+ T cells, apoptosis was significantly increased, indicating that MyD88 supports CD8+ T cell survival post-activation (17). Despite these defects, MyD88 was not required for graft-versus-leukemia activity, suggesting that MyD88 regulates effector function and survival without altering TCR-mediated tumor recognition (17). Together, these studies highlight the MyD88/IRAK4 axis as a potential target for modulating CD8+ T cell activation and effector responses irrespective of TLR stimulation.

### TCR-independent TLR-mediated activation of CD8+ T cells

Traditionally, CD8+ T cell activation is strictly dependent on antigen recognition through the TCR. However, recent studies have demonstrated that subsets of CD8+ T cells can directly sense TLR ligands, leading to the production of IFN-γ and other cytokines without antigen-specific stimulation (18–20). This process of innate activation is particularly evident in innate-like CD8+ memory T cells. Unlike conventional CD8+ memory T-cells (T_MEM_ cells), which develop following antigen encounter, innate-like CD8+ T_MEM_ cells arise during thymic development and are pre-programmed to rapidly produce cytokines like IFN-γ in response to innate immune signals (18, 21–24). In 2015, Ghosh et al. demonstrated the role of TLR4 in modulating the cytokine response of innate-like CD8+ T_MEM_ cells in mice (18). The study found that these cells constitutively expressed high levels of TLR4 and produced IFN-γ and IL-2 (18). Stimulation with LPS upregulated IFN-γ while suppressing IL-10 via SOCS1 induction, skewing the innate-like CD8+ T_MEM_ cells towards a proinflammatory phenotype (18). In contrast, heat shock protein 70 (Hsp70) downregulated TLR4 to promote IL-10 secretion and induced PD-1 expression, shifting the cells towards immunosuppressive function (18). Similarly, Rubtsova et al. (19) demonstrated that innate-like CD8+ T_MEM_ cells can produce IFN-γ in response to TLR7 signaling, independent of TCR engagement. This process was IL-12 and MyD88 dependent (19). The study found that CD8+ central T_MEM_ cells (CD44+CD62L+) responded to TLR7 stimulation by producing IFN-γ, whereas naïve CD8+ T cells did not (19). Mechanistically, the authors found that MyD88-deficient CD8+ T cells failed to produce IFN-γ upon TLR7 activation, confirming that TLR7 signaling was intrinsic to CD8+ T cells (19). Collectively, these findings underscore the capacity of TLR-mediated signaling to directly stimulate innate-like CD8+ T_MEM_ cells, enabling rapid, TCR-independent effector responses that bridge both innate and adaptive immunity.

In cancer, chronic antigen stimulation through the TCR is known to drive CD8+ T cell exhaustion, rendering these cells dysfunctional and unable to control disease progression. As such, understanding these TLR-mediated noncanonical activation pathways may open new avenues for cancer immunotherapies to overcome TCR-dependent mechanisms of dysfunction in CD8+ T cells. O’Donnell et al. (25) demonstrated that CD8+ T cells can be activated independently of TCR engagement through TLR and inflammasome signaling, enhancing effector function during bacterial infection (25). Specifically, the study found that CD8+ T cells rapidly secreted IFN-γ in response to TLR2, TLR4, and TLR5 ligands, even in the absence of direct antigen recognition (25). Importantly, this response was dependent on CD8+ T cell-intrinsic MyD88 signaling, as MyD88 deficiency completely abrogated IFN-γ production (25). Salerno et al. (20) then corroborated these findings by demonstrating that CD8+ T cells can directly sense TLR2 and TLR7 ligands, leading to the rapid production of IFN-γ without TCR engagement (20). Mechanistically, IFN-γ production was regulated by the PI3K-AKT signaling pathway, and protein synthesis depended on mTOR (20). Unlike antigen-specific activation, which necessitates glycolysis for cytokine production, TLR-driven IFN-γ release by CD8+ T cells relied on mitochondrial respiration for energy (20). Together, these insights highlight the paradigm in which CD8+ T cells integrate TLR signaling to modulate effector function independently of classical antigen-dependent TCR responses. Future research may investigate TLR-dependent, TCR-independent avenues to boost tumor-specific CD8+ T cell effector activity.

Together, these studies have highlighted an underappreciated mechanism of TLR signaling in CD8+ T cell activation, independent of TCR engagement. This TLR-driven response, seen in both innate-like CD8+ T_MEM_ and antigen-experienced CD8+ T cells, enhances cytokine production, allowing these cells to rapidly respond to infection. Of note, this pathway also poses challenges in autoimmune diseases such as Rheumatoid Arthritis, where aberrant TLR signaling in CD8+ T cells may contribute to chronic inflammation (26, 27). As such, understanding these alternative innate activation mechanisms is critical for developing therapeutic strategies aimed at modulating CD8+ T cell function in both antitumor immunity and immune-mediated pathologies.

### TLR-based therapeutics in cancer immunotherapy

TLR-based therapeutics have been explored for decades, initially focusing on their role as vaccine adjuvants (28), with later applications in cancer and autoimmune diseases (29, 30). However, while TLR agonists can enhance CD8+ T cell-mediated antitumor responses, cancer cells and other immunosuppressive subsets also often express TLRs, where signaling can paradoxically support tumor progression (30). As such, significant efforts have been directed towards optimizing TLR-based therapeutics to preferentially enhance antitumorigenic effects in CD8+ T cells. For example, Lynn et al. (31) introduced a self-assembling nanoparticle vaccine (SNP-7/8a) that enhanced CD8+ T cell priming by linking peptide neoantigens to TLR7/8 agonists (31). With SNP-7/8a-mediated uptake and MyD88-dependent APC activation, the conjugated neoantigens were better presented to prime CD8+ T cells, increasing expression of activation markers (CD44, CD69, and CD25) and cytokine production (IFN-γ, TNF-α), culminating in a more robust CD8+ T cell antitumor response, with demonstrated translational potential in non-human primates (31). Similarly, a biodegradable nanoparticle platform delivering IL-2, anti-CD28, Pam3CSK4, and a NOD2 agonist, was shown to stimulate endogenous tumor-infiltrating T cells and slow tumor growth (32). These advances in targeted delivery systems offer a strategic means to harness the immunomodulatory effects of TLR agonists while minimizing pro-tumorigenic off-target effects. Manna et al. (33) further corroborated these findings by covalently linking TLR2/6 and TLR7/8 agonists to pathogen-like nanoassemblies (MTAs), which remarkably enhanced CD8+ T cell mediated antitumor responses with low off-target toxicity in murine melanoma; however, this study did not report if the TLR stimulus was acting on APCs or CD8+ T cells (33). As such, we continue by examining cancer-specific applications of TLR-based therapeutics that directly signal in CD8+ T cells, the ongoing challenges, and new strategies to optimize their efficacy while mitigating associated risks.

While TLR stimulation in CD8+ T cells has been shown to bolster antitumor responses, transient expression of TLRs on CD8+ T cells and difficulties with agonist infiltration into solid tumors, have narrowed the therapeutic potential of TLR agonists in cancer. An alternative strategy is to selectively induce TLR signaling in antitumor CD8+ T cells through direct genetic modifications that confer intrinsic TLR activity in adoptive cell therapy products. For example, Foster et al. (34) and Collinson-Paultz et al. (35) investigated the use of a MyD88/CD40 (MC) co-stimulatory domain to improve CAR-T cells therapy (34, 35). Foster et al. developed an inducible MyD88/CD40 (iMC) system, where co-stimulation was triggered by the small molecule rimiducid, allowing for controlled expansion and cytotoxicity of CAR-T cells while minimizing systemic toxicities (34). In contrast, Collinson-Paultz et al. (35) engineered a constitutively active MC system, resulting in enhanced CAR-T cell proliferation and antitumor activity (35). However, continuous co-stimulation resulted in severe cytokine release syndrome-like toxicities, and an inducible caspase-9 (iC9) safety switch was incorporated to selectively deplete CAR-T cells without compromising efficacy (35). In 2020, Prinzing et al. (36) then expanded on these findings by demonstrating that MyD88/CD40 signaling preserved CAR-T cells in a less differentiated state, enhancing their functional longevity and persistence compared to CD28- and 41BB-CAR T cells (36). In murine osteosarcoma models, EphA2-targeting MC-CAR T cells retained a central memory phenotype (CCR7+CD45RA−) with reduced expression of differentiation-associated transcription factors T-bet and BLIMP1 compared to CD28- and 41BB-CAR T cells (36). Mechanistically, MC-CAR T cells expressed higher levels of MYB and FOXM1, key regulators of cell cycle progression and self-renewal, and upregulated pathways essential for T cell metabolism(mTORC1 and MYC) (36). Functionally, MC-CAR T cells exhibited prolonged expansion, cytotoxicity, and lower susceptibility to activation-induced cell death (36). These findings highlight MyD88/CD40 signaling as a strategy to maintain CAR-T cell fitness, improving therapeutic efficacy while managing toxicity as compared to conventional second-generation CAR design.

An alternative approach to harness TLR/MyD88 signaling in CD8+ T cells was demonstrated by Kaczanowska et al. (37). The study presented the enhanced antitumor efficacy against weakly immunogenic tumor antigens by CD8+ T cells engineered with a synthetic CD8α:MyD88 fusion protein (37). By fusing the extracellular domain of CD8α to the MyD88 signaling domain, they created a construct that activated MyD88 signaling in a TCR-dependent but TLR-independent manner (37). CD8α:MyD88 T cells exhibited enhanced proliferation, cytokine production (IFN-γ, TNF-α), elevate cytotoxic activity, and increased costimulatory receptor expression, leading to tumor regression in a B16-F1 melanoma model (37). Mechanistically, CD8α:MyD88 signaling increased ZAP-70, ERK1/2, JNK, and p38 activation, reducing the response threshold in tumor-specific CD8+ T cells (37). Subsequently, Ciavattone et al. (38) investigated the impact of MyD88 co-stimulation in donor CD8+ T cells within the context of allogeneic hematopoietic cell transplantation (allo-HCT) for hematological malignancies (38). They demonstrated that stimulation of CD8+ T cells through the CD8α:MyD88 construct improved control of A20 lymphoma in mice (38). This enhanced graft-versus-tumor (GVT) effect was a result of increased T cell expansion, cytokine production, and cytotoxic killing of tumor cells (38). However, MyD88 co-stimulation also led to increased, yet non-lethal, graft-versus-host disease (GvHD), indicated by weight loss and inflammation (38). These findings underscore the power of TLR-based co-stimulation in adoptive T cell therapies but highlight the need for precise modulation to balance efficacy and safety in clinical applications.

In **Table 1**, we provide a summary of TLR agonist therapies and their functional consequences in antitumor immunity. These studies collectively highlight the versatility of TLR-based strategies for enhancing antitumor immunity across various therapeutic platforms, including ICI, nanoparticle vaccines and adoptive cell therapies.

**Table 1:** Summary of TLR signaling consequences in CD8+ T cells and MyD88-based cellular immunotherapies.

TLR Complex	Natural Ligand(s)		Synthetic Ligand(s)/Mimetics	Adaptor Molecule(s)	Functional Roles in CD8+ T cells	References
TLR1/2	Triacyl-lipopeptides		Diprovocim, Pam3CSK4	MyD88	Enhances activation, expansion, and cytokine production (IFN-γ, IL-2) Upregulates metabolic regulators (IRF4, BATF, T-bet) Drives metabolic reprogramming towards increased glycolysis, glucose uptake, and glutaminolysis Drives metabolic reprogramming towards increased glycolysis, glucose uptake, and glutaminolysis Elevates mitochondrial respiration and ATP production Promotes expression of costimulatory surface molecules (41BB, OX40, GITR, LTA) Enhances NF-κB (p65) and AP-1 (c-Jun) binding to the 41BB promoter Supports both de novo transcription and mRNA stability of cytokine genes, prolonging IFN-γ mRNA half-life and enhancing polyfunctionality	(11) Joseph et al. (2016) (12) Zhang et al. (2019) (14) Imanishi et al. (2020) (15) Salerno et al. (2019) (39) Wang et al. (2018)
TLR2/6	Diacyl-lipopeptides, FSL-1, Zymosan		Pathogen-like nanoassemblies (MTAs) of TLR2/6 agonists	MyD88	FSL-1 enhances IFN-γ production (dependent on TIRAP expression induced by TCR and IL-2 mediated mTORC1 signaling) Zymosan increases IFN-γ transcription, but NOT protein expression MTAs of TLR2/6 agonists enhance CD8+ T cell mediated antitumor responses	(14) Imanishi et al. (2020) (28) Manna et al. (2020)
TLR3	dsRNA		Poly(I:C)	TRIF	TRIF alone promotes type-I IFN signaling and strengthens CD8+ T cell priming, activates NF-κB and IRF3, induces DAMP release and necroptotic tumor cell death Co-administration of TLR3 agonists with anti-CTLA-4 or anti-LAG-3 enhances vaccine-mediated tumor suppression	(32) Jeon et al. (2024)
TLR4	Lipopolysaccharides (LPS)		Monophosphoryl lipid A (MPLA)	MyD88, TRIF	In innate memory CD8+ T cells, LPS upregulates IFN-γ and suppresses IL-10 In antigen-experienced CD8+ T cells, ligands can induce rapid IFN-γ secretion in a TCR-independent manner, requiring T cell-intrinsic MyD88 signaling and IL-18R expression In RA-derived CD8+ T cells, aberrant TLR4 expression leads to direct activation by LPS, upregulating cytotoxic molecules (granzyme B, perforin) and inflammatory cytokines (TNF-α, IFN-γ) independent of TCR engagement	(18) Ghosh et al. (2015) (25) O'Donnell et al. (2014) (26) Tripathy et al. (2017)
TLR5	Flagellin			MyD88	TCR independent secretion of IFN-γ	(25) O'Donnell et al. (2014)
TLR7	ssRNA			MyD88	Co-stimulation promotes cellular glycolysis, enhancing effector function, leading to increased expression of glycolytic enzymes (Glut1, HK2, LDHa) and higher IFN-γ, TNF-α, and IL-2 production IRF4 links TLR7-driven metabolic reprogramming to effector function Promotes IFN-γ production independent of TCR engagement in memory CD8+ T cells	(19) Rubstova et al. (2016) (13) Li et al. (2019)
TLR7/8	ssRNA		R848, Pathogen-like nanoassemblies (MTAs) of TLR7/8 agonists	MyD88	Agonists linked to peptide antigens in self-assembling nanoparticle vaccines (SNP-7/8a) enhanced CD8+ T cell immunity against tumor neoantigens, improved antigen uptake by DCs, and enhanced CD8+ T cell priming SNP-7/8a peptides directly stimulated CD8+ T cells, synergizing with TCR activation to increase expression of activation markers and cytokine production in a MyD88-dependent manner MTAs of TLR7/8 agonists enhanced CD8+ T cell mediated antitumor responses.	(31) Lynn et al. (2020) (33) Manna et al. (2020)
TLR9	CpGA, CpGB		ODN1826	MyD88	Promotes tumor clearance by enhancing CD8+ T cell activation and antigen presentation Inhaled agonists enhance efficacy of PD-1 blockade in lung tumors, remodeling the TME, enhancing CD8+ T cell infiltration, dendritic cell activation, and expansion of PD-1(low) T-bet(high) effector CD8+ T cell Co-administration with TLR3 agonists and anti-CTLA-4 or anti-LAG-3 enhanced vaccine-mediated tumor suppression	(40) Gallotta et al. (2018) (41) Jeon et al. (2024)
Cell Therapy	Modifications			Functional Outcomes		References
iMC CAR T-cell	Extracellular: CD19 scFv Intracellular: CD40, MyD88, FKBP12v36 (rimiducid receptor)			Upon addition of rimiducid, a lipid-permeable dimerizing ligand, CD40 and MyD88 are activated to induce effector cytokine production (GM-CSF, IFN-γ, TNF-α, IL-2) Antigen-independent enhanced survival and proliferation Improved tumor control		(34) Foster et al. (2017)
iC9-CD19.z-MC CAR T-cell	Extracellular: CD19 scFv Intracellular: CD40, MyD88, CD3z Co-expressed with FKBP-Casp9			Enhanced proliferation, survival, effector cytokine production, persistence and tumor control Retains less differentiated T-cell state Increased expression of MYB and FOXM1 Upregulated mTORC1 and MYC pathways Inducible apoptosis driven by rimiducid binding to FKBP-Casp9 to abbrogate systemic toxicities		(35) Collinson-Pautz et al. (2019) (36) Prinzing et al. (2020)
CD8α:MyD88 T-cell	Extracellular: CD8α Intracellular: MyD88			Reduced antigen response threshold Decreased expression of T cell exhaustion markers Enhanced proliferation, survival, effector cytokine production, costimulatory molecule expression, cytotoxicity and tumor control		(30) Kaczanowska et al. (2017) (38) Ciavattone et al. (2021)

### Challenges and future directions of TLR-based therapeutics

The role of TLR signaling in CD8+ T cells extends beyond its classical function in innate immunity, playing a crucial role in shaping the adaptive antitumor response. TLR engagement can enhance TCR-mediated activation, promote metabolic reprogramming, and facilitate costimulatory receptor upregulation, thereby augmenting CD8+ T cell expansion and effector function (11–15, 19, 25). Additionally, certain TLR pathways can bypass traditional antigen recognition, enabling TCR-independent activation, which is particularly relevant in chronic infections, cancer immunotherapy, and autoimmunity (18–20, 25, 26).

In cancer, TLR agonists have been therapeutically explored as immune modulators to overcome suppressive tumor microenvironments, enhance the efficacy of ICIs, and improve CAR-T cell function (34–37, 39–42). Despite their potential, several challenges must be addressed for TLR-based therapeutics to achieve widespread clinical application. Balancing enhanced CD8+ T cell activation while minimizing systemic toxicity remains a significant hurdle, requiring strategies such as nanoparticle-based delivery systems and cell-targeted approaches. Specifically, optimizing the clinical utility of TLR-based immunotherapies will require a deeper mechanistic understanding of how TLR signaling operates within CD8+ T cells. Future studies may investigate the crosstalk between TLR signaling and costimulatory/coinhibitory receptor signaling pathways to guide therapeutic development and combination approaches with ICIs.

Similarly, an important frontier for future research lies in dissecting the impact of TLR signaling on noncanonical signaling cascades in CD8+ T cells. For example, increasing evidence supports the importance of noncanonical NF-kB activity in sustaining CD8+ T cell survival, persistence and memory formation – features essential for durable antitumor immunity (43–46). Although TLRs have been shown to trigger noncanonical NF-κB signaling in other immune contexts (47–49), this axis remains largely unexplored within CD8+ T cells. Elucidating this mechanistic link could reveal novel regulatory mechanisms and provide a conceptual framework for engineering TLR-based therapies that enhance the durability of antitumor responses.

## Cytosolic nucleic acid sensors: STING and RLR pathways

In addition to endosomal TLRs, innate immune cells rely on other cytoplasmic nucleic acid sensors to detect foreign or aberrant self-DNA and RNA. Two such families include the Stimulator of Interferon Genes (STING) and RIG-I-like Receptor (RLR) families. Herein, we discuss the recent advancements in exploiting nucleic acid sensing pathways to augment CD8+ T cell antitumor immunity and therapeutic efficacy.

### STING and RLR nucleic acid sensors are essential to immunosurveillance

**Figure 2:**
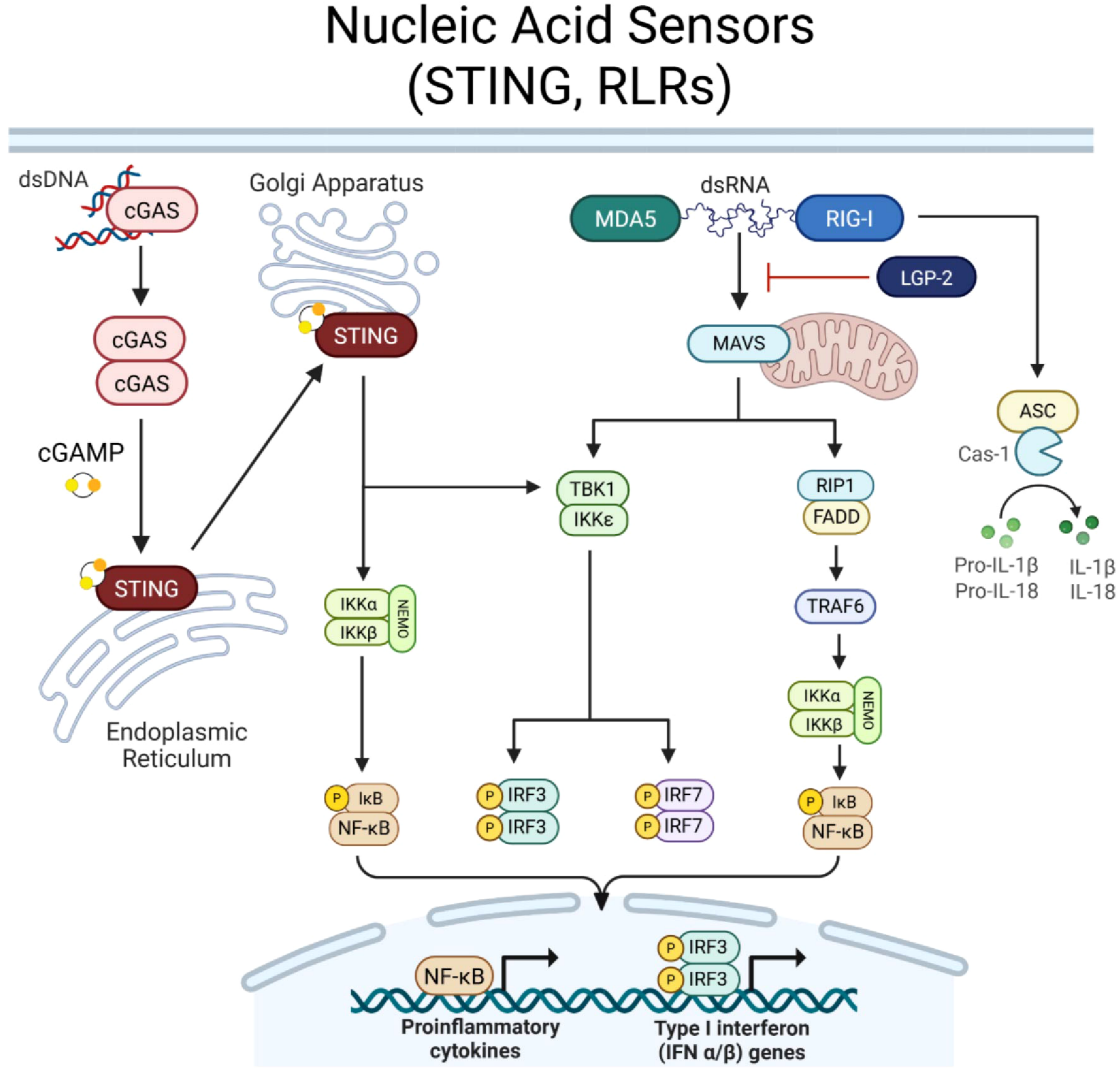
Nucleic acid sensor signaling. Cyclic GMP-AMP synthase (cGAS) recognizes cytosolic double stranded (ds)DNA. Upon binding, cGAS dimerizes and synthesizes secondary messenger cyclic GMP-AMP (cGAMP). STING is located in the membrane of the Endoplasmic Reticulum and upon interaction with cGAMP, STING is activated and translocates to the Golgi Apparatus. STING proceeds to induce NEMO complex activation and phosphorylation of IkB to induce NF-kB transcriptional activity. STING can also induce TBK1, resulting in phosphorylation of IRF3/7. RIG-I-like receptors, RIG-I and MDA5, recognize cytosolic dsRNA molecules, while third family member, LGP-2, acts to regulate RIG-I/MDA5 signaling. Upon dsRNA binding, RIG-I/MDA5 activate mitochondrial protein, MAVS, which transmits signal to activate (1) TBK1, inducing IRF3/7 activity, or (2) RIP1, which recruits FADD to activate TRAF6 and thus, the NEMO complex to activate NF-kB. Together, STING and RLR signaling promote NF-kB and IRF3/7 transcriptional activity to induce a proinflammatory and type-I IFN immune response. RIG-I can also bypass MAVS and directly promote caspase-1 activity, cleaving pro-forms of IL-1β and IL-18 to drive proinflammatory cytokine release. Created with BioRender.com.

In the membrane of the endoplasmic reticulum (ER), STING serves as a key mediator of cytosolic DNA sensing (**Figure 2**). Its activation is triggered by secondary messenger 2’3’ cyclic guanosine monophosphate–adenosine monophosphate (cGAMP) (2, 50–53). cGAMP is synthesized by DNA sensor, cyclic GMP-AMP synthase (cGAS), upon binding to double-stranded DNA in the cytosol (2, 50–53). cGAMP causes STING to undergo a conformational change, facilitating STINGs translocation from the ER to the Golgi Apparatus (51, 53). In the Golgi, STING forms a complex with TANK-binding kinase 1 (TBK1) and inhibitor of kappa B kinase (IKK), leading to phosphorylation and activation of key transcription factors, including IRF3 and NF-κB (2, 50–53). These downstream effectors drive the production of type I interferons (IFN-α, IFN-β) and proinflammatory cytokines. In addition to sensing pathogen-derived cytosolic DNA, STING also responds to cyclic dinucleotides (CDNs), bacterial byproducts, and signals associated with genomic instability, reinforcing its role in immune surveillance beyond infection (2, 50–53).

While STING is dedicated to DNA sensing, RLRs specialize in detecting cytosolic RNA species (**Figure 2**). The RLR family comprises three key members: (1) RIG-I (Retinoic acid-inducible gene I, DDX58), which recognizes short double-stranded RNA (dsRNA) and RNA with a 5’ triphosphate, (2) MDA5 (Melanoma differentiation-associated protein 5, IFIH1) which detects long dsRNA, and (3) LGP2 (Laboratory of Genetics and Physiology 2, DHX58) which primarily functions as a regulatory cofactor, modulating RIG-I and MDA5 signaling (2). RIG-I and MDA5 activation is initiated by ligand binding at the C-terminal domains, which induces a conformational change to expose the N-terminal caspase activation and recruitment domain (CARD) (2). The CARDs interact with the mitochondrial antiviral signaling protein (MAVS), which serves as a scaffold for downstream signaling (2). MAVS recruits TRAF family proteins and activates TBK1 and IKK, leading to phosphorylation of IRF3, IRF7, and NF-κB, culminating in type I IFN production and inflammatory cytokine release (2). Moreover, RIG-I can bypass MAVS to induce caspase-1 activity to cleave pro-forms of inflammatory cytokines, IL-1β and IL-18, and facilitate their release (2).

Only in the last decade has research focused on the effects of CD8+ T cell-intrinsic STING activation. A pivotal study in 2017 by Poltorak provided the first direct evidence that STING is functionally active in CD8+ T cells (52). In this study, short or low level STING activation in T cells modulated their function in a manner distinct from its role in innate immunity, revealing STING’s ability to influence T cell survival and effector responses (52). Importantly, prolonged or high-level STING activation seemed to be detrimental, leading to endoplasmic reticulum (ER) stress, unfolded protein response (UPR), and even apoptosis of T cells. Unlike STING, there is no clear consensus on the first study to describe RLR signaling in T cells. However, studies from the 2010s confirmed RIG-I expression and its intrinsic signaling effects in CD4+ T cells, suggesting a broader role for RLRs in T cell immunity (54). These discoveries have sparked ongoing research into the functional consequences of STING and RLR activation in adaptive immune cells. Understanding how these pathways integrate with TCR signaling, persistence, and differentiation may provide new therapeutic strategies to enhance T cell responses.

### Balancing STING activation in CD8+ T cells: effector function vs. apoptosis

Unlike innate immune cells, where STING primarily drives a type I IFN response, T cell-intrinsic STING activation elicits distinct outcomes that are dependent on the timing and strength of signal. Studies have demonstrated that low levels of STING activation enhance T cell effector function, while high levels inhibit proliferation and drive apoptosis (50, 52, 55–60), highlighting a critical threshold that determines the impact of STING activity on T cells.

Early findings by Larkin et al. (52) revealed that synthetic STING agonists, such as DMXAA, could promote type-I IFN production in both CD4+ and CD8+ T cells (52). However, high doses of DMXAA early after TCR activation impaired proliferation and prompted T cell death (52). RNA sequencing identified increased expression of interferon-stimulated genes (ISGs) alongside markers of caspase-mediated apoptosis, endoplasmic reticulum (ER) stress, and the unfolded protein response (UPR) (52). Wu et al. (58) supported these findings by mechanistically linking STING activation with ER stress and the UPR (58). The study demonstrated that constitutively active STING (Sting^N153S^) disrupted calcium homeostasis which rendered T cells hyperresponsive to TCR signaling, resulting in ER stress, UPR activation, and ultimately inducing apoptosis (58). Interestingly, a novel “UPR motif” in the STING protein was identified to be required for this effect and this motif was distinct from domains required for type-I IFN responses (58). Further studies by Gulen et al. reinforced the concept that STING signaling strength dictates distinct outcomes in T cells (59). Using the STING agonist CMA, researchers observed that while STING activation upregulated antiviral genes in CD8+ T cells, its predominant effect was apoptosis, a stark contrast to the apoptosis-resistant response seen in APCs (59). This apoptotic response was attributed to high STING expression in T cells, leading to amplified signaling and activation of IRF3 and p53-dependent proapoptotic pathways (59). Notably, this mechanism was also functional in malignant T cells, where pharmacological hyperactivation of STING effectively suppressed T cell-derived cancers, including T-ALL and T cell lymphoma (59). These findings established that STING’s proapoptotic function is determined by the magnitude of its activation, presenting a potential avenue for targeted therapies in T cell malignancies. How or whether STING-mediated T cell death within a tumor can contribute to developing an inflammatory response that improves recruitment and activation of other immune cells has yet to be explored, but merits further consideration.

The use of different synthetic ligands to modulate STING signal strength has emerged as a promising strategy for fine-tuning CD8+ T-cell responses. Quaney et al. (61) clearly demonstrated this effect using engineered *Listeria monocytogenes* (LM) strains producing varying levels of STING agonist, c-di-AMP (61). High STING activity impaired the differentiation of OTI memory CD8+ T cells after acute LM-OVA infection by upregulating the proapoptotic factor Bim (61). Interestingly, other studies have indicated that low-dose STING agonists such as ADU-S100 (S100), DMXAA, and the STING^V155M^ mutation, can enhance antitumor CD8+ T cell responses without compromising viability (55–57, 62, 63). Lv et al. (62) revealed that cGAS agonist Manganese can enhance cytotoxic T lymphocyte (CTL) activity and CD8+ T cell memory differentiation, leading to sustained antitumor immunity in solid tumor models (62). Likewise, Wang et al. showed that administration of STING agonist diABZI compound 3 (C3) enhanced antitumor efficacy of transferred T cells in melanoma models (57). Specifically, diABZI enhanced TCR signaling as evidenced by increased phosphorylation of ZAP-70 and p38, increasing T cell cytotoxicity to Mel526 tumors and prolonging IFN-γ production (57). Collectively, these studies present the potential for fine-tuning strength of CD8+ T-cell intrinsic STING signaling strength to amplify antitumor responses. However, more work is needed to understand the effects of signal duration on dictating specific outcomes in CD8+ T-cells.

In addition to STING dosage, TCR signal strength can also modulate the impact of STING on CD8+ T cell survival and memory differentiation. Wu et al. (58) showed that constitutively active STING (STING^N153S^) induced an elevated sensitivity to TCR engagement, causing chronic ER stress and activation induced T cell death (58). Building on this idea, Quaney et al. (61) found that T cells receiving both strong TCR and STING signals exhibited impaired memory differentiation during the contraction phase of infection, while low-affinity memory T cells receiving high dose STING stimulation displayed enhanced recall abilities in both infection and tumor models (61). Mechanistically, high-affinity CD8+ T cells exposed to STING agonists showed greater phosphorylation of STING, IRF3, and TBK1, leading to activation of the UPR and promoting apoptosis (61). The key regulator of this differential response was the transcription factor C/EBP homologous protein (CHOP), which participates in UPR-mediated apoptosis through Bim upregulation (61). In low-affinity CD8+ T cells, STING activation resulted in reduced CHOP expression, thereby supporting survival and memory differentiation (61). These findings suggest that STING activation, when carefully calibrated to TCR strength, can enhance T cell function while preventing excessive cell death. This balance holds therapeutic potential, especially in tumor immunotherapy, where low-affinity CD8+ T cells specific for weakly immunogenic tumor antigens may benefit from STING-mediated enhancement of effector function.

The functional importance of STING in CD8+ T cells is further demonstrated by studies showing that complete loss of STING impairs antitumor immunity. In 2020, Li et al. found that CD8+ T cells from cancer patients exhibited significantly reduced cGAS-STING expression compared to healthy individuals (64). Further, knockout of cGAS-STING in CD8+ T cells accelerated tumor progression, reduced T cell proliferation and cytokine production, and diminished expression of stemness markers, TCF1 and SLAMF6 (Ly108) (64). Mechanistically, STING promoted stem-like differentiation by inducing autocrine type I IFN signaling, which inhibited chronic AKT activation, a known driver T cell dysfunction (64). Accordingly, STING agonism in tumor-specific CD8+ T cells favored the expansion of stem-like memory (T_SCM_) populations over terminally exhausted T cells (64). In support of this relationship between STING activity and stemness, recent studies have identified STING as a key regulator of stem-like TCF1+ NK cells during antitumor responses. Lu et al. demonstrated that conditional deletion of STING in NK cells impaired their effector functions and downregulated TCF1, suggests a broader role for STING in maintaining long-term immune function and potential for a novel strategy to expand TCF1+ NK cells (65). Together, these findings suggest that STING signaling can be leveraged to maintain CD8+ T cell and NK cells stemness, supporting its therapeutic potential further investigations to synergize with ICIs.

Emerging evidence also suggests that STING can influences CD4+ T cell fate to augment antitumor immunity. In 2022, Benoit-Lizon et al. revealed that STING activation modulates CD4+ T cell differentiation between Th1 and Th9 subsets through distinct mechanisms (66). Th1 differentiation was driven by canonical STING signaling via IRF3 and type I IFN induction, whereas Th9 skewing occurred independently of IFN signaling and instead relied on STING-mediated activation of mTOR effector pathways (66). Notably, the degree of STING activation dictated this divergence, as Th1 cells were more sensitive to STING ligand-induced apoptosis than Th9 cells (66). Given the growing interest in Th9-mediated antitumor responses, these findings suggest a potential strategy for leveraging STING agonists to bolster Th9 differentiation and enhance therapeutic efficacy beyond classical CD8+ T cell-based approaches.

Collectively summarized in **Table 2**, these studies establish that STING signaling in T cells must be finely tuned to balance effector function and survival. Strong STING activation signals may drive apoptosis, particularly in high-affinity T cells, whereas moderate to low STING signaling supports memory differentiation, stemness, and enhances antitumor immunity. These insights underscore the potential of STING-targeted therapies in shaping T cell responses in infection, cancer, and immunotherapy.

**Table 2:** Summary of STING influence on CD8+ T cells in antitumor immunity.

Agonist(s)	Mutation(s)	Functional Roles in CD8+ T cells	References
DMXAA, Manganese, ADU-S100, diABZI-C3	STING^V155M^	STING activation enhanced TCR signaling through phosphorylation of ZAP-70 and p38 resulting in increased T cell cytotoxicity Low doses of STING agonist supported T cell tumor infiltration and IFN-γ production	(56) Sivivk et al. (2018) (57) Wang et al. (2024) (62) Lv et al. (2020) (63) Tse et al. (2021)
DMXAA, CMA, c-di-AMP	STING^N153S^ STING^V155M Δ368^ STING^V155M Δ354^ STING^V155M S366A^	High doses of STING agonist or STING stimulation can drive T cell death vias caspase-mediated apoptosis High levels of STING expression induced p53-dependent proapoptotic mechanisms in T cells through upregulation of *Puma, Noxa*, and *Bim* Mutations that cause constitutive activity of STING can interfere with calcium homeostasis, driving ER stress and the UPR to induce T cell apoptosis Activating mutations in STING inhibited T cell proliferation	(52) Larkin et al. (2017) (59) Gulen et al. (2017) (60) Cerboni et al. (2017) (61) Quaney et al. (2023) (58) Wu et al. (2019)
c-di-AMP	STING^N153S^	High affinity TCR/ high dose STING impaired memory differentiation by activating the UPR and driving apoptosis via upregulation of CHOP Low affinity TCR/ high dose STING enhanced memory T cell recall responses by reducing CHOP expression and thus Bim-mediated apoptosis Constitutive STING activity induced hypersensitivity to TCR stimulation, resulting in apoptosis	(61) Quaney et al. (2023) (58) Wu et al. (2019)
diABZI-C3		Depletion of STING in CD8+ T cells reduced effector cytokine production STING promotes stem-like CD8+ T cell differentiation by inhibiting chronic Akt activation	(64) Li et al. (2020)
DMXAA		Increased CAR-T cell persistence Increased sensitivity of T cells to checkpoint blockade	(61) Quaney et al. (2023) (64) Li et al. (2020)

### Effects of RIG-I on tumor-specific T cells

Given the parallelisms between STING and RLR signaling pathways, research into the T cell intrinsic effects of RIG-I similarly highlight the dichotomy of responses. Emerging evidence underscores the role of RIG-I (Ddx58) as a critical regulator of CD8+ tumor-infiltrating lymphocyte (TIL) function, implicating RIG-I as an immune checkpoint (67, 68). Jiang et al. (69) demonstrated that RIG-/- CD8+ T cells exhibited elevated levels of anti-apoptotic proteins (BCL-2/BCL-xL) and reduced levels of pro-apoptotic markers (Annexin V, BAX, and cleaved caspase-3) (69). Functionally, RIG-I deficiency increased in effector molecule expression in CD8+ TILs and shRNA knockdown of RIG-I in CD19-CAR-T enhanced survival and cytotoxic function against Nalm6 leukemia cells (69). Transcriptomic analysis of RIG-/- CD8+ T cells revealed upregulation of effector genes, downregulation of inhibitory receptors, and Gene Set Enrichment Analysis (GSEA) highlighted IL-2-STAT5 signaling as a key pathway enriched by the loss of RIG-I. Increased STAT5 and STAT3 phosphorylation was observed in RIG-/- CD8+ T cells upon activation, and co-immunoprecipitation revealed direct RIG-I/HSP90 interactions, with RIG-I deficiency enhancing HSP90-STAT5 association (69). Together, these findings suggest that RIG-I may oppose CD8+ T cell survival and function through regulation of STAT5 signaling, supporting its role as a checkpoint-like regulator of antitumor immunity.

Recently, Duan et al. (68) published a comprehensive analysis of several public single-cell RNA sequencing (scRNA-seq) datasets of tumor tissues (68). The authors found that exhausted CD8+ TILs—characterized by the upregulation of *Pdcd1, Entdp1*, and *Tigit*—also exhibited increased RIG-I expression, while other innate immune receptors and co-factors such as STING, MDA5, and MAVS remained unchanged, suggesting a selective role for RIG-I in TIL biology (68). Functional studies confirmed that RIG-/- CD8+ T cells displayed elevated expression of cytolytic (perforin, granzyme B) and effector (IFN-γ, TNF-α) molecules (68). Notably, immunodeficient B-NDG mice with immortalized lymphoblastoid cell line (LCL) tumors treated with RIG-I shRNA knockout human CD8+ T cells exhibited delayed tumor progression, with increased frequencies of cytolytic TIL (68). Similarly, adoptive transfer of RIG-/- T cells into MC38 tumor-bearing mice significantly impaired tumor growth (68). Mechanistically, RIG-/- CD8+ T cells exhibited increased AKT phosphorylation, suggesting that RIG-I may act as a negative regulator of AKT signaling (68). AKT phosphorylation is a key metabolic checkpoint downstream of RIG-I activation and is also essential for CD8+ T cell metabolism and function. While additional studies are necessary to explicitly delineate this regulatory axis, these findings provide a foundation for further investigation into the role of RIG-I in shaping CD8+T cell antitumor responses.

In 2021, Johnson et al. sought to improve CAR T-cell therapies by leveraging endogenous RNAs, such as RN7SL1, to activate RIG-I/MDA5 signaling (70). RN7SL1 is a highly structured noncoding RNA conserved across species that mimics viral RNA to selectively activate RIG-I in immune cells (70). In a pancreatic xenograft model, RN7SL1-expressing CAR-T cells exhibited enhanced antitumor efficacy with greater expansion and persistence of RN7SL1 CAR-T cells with an effector-memory phenotype (70). Notably, RN7SL1 increased the functional longevity of CAR-T cells and sustained tumor clearance, which was not seen in systemic RN7SL1 delivery, indicating that tumor response depended on CAR-target interactions (70). scRNA-seq revealed that RN7SL1 CAR-T cells maintained a memory-like transcriptome, whereas control CAR-T cells appeared exhausted, expressing higher levels of *Pdcd1*, *Tigit*, and *Tox* (70). Notably, beyond the intrinsic CAR-T cell benefits, RN7SL1 was exported via extracellular vesicles, selectively activating type I IFN signaling in other immune cells (70). This process reshaped the TME by restricting suppressive myeloid cell features, increasing the expression of costimulatory ligands on DCs, and supporting endogenous CD8+ T cell priming and recruitment to tumors (70). Importantly, these stimulatory effects of RN7SL1 were lost in MAVS- or RIG-I-deficient bone marrow-derived cells, confirming dependence on RIG-I signaling (70). Together, the expression of RN7SL1 in CAR-T cells enhanced persistence, cytotoxicity, and reshaped the TME. Despite these studies, the specific mechanisms by which RIG-I activity influence CD8+ T cell differentiation and cytotoxicity remain poorly understood, with most research focusing on the intrinsic role of RIG-I in cancer cells or APCs, and indirect roles in bolstering CD8+ T cell responses. As RLR and STING signaling pathways overlap, future investigations may seek to compare/contrast the functional outcomes of these nucleic acid sensors as it relates to CD8+ T cell antitumor immunity and therapeutic development for cancer.

Summarized in **Table 3**, these findings suggest that RIG-I can function as a regulator of CD8+ T cell fate, balancing survival, effector function, and metabolic signaling in a context/stimulation dependent manner. By restraining STAT5 activation through HSP90 sequestration, RIG-I appears to limit cytotoxic T cell expansion while promoting apoptotic pathways, ultimately dampening antitumor immunity. However, the translational relevance of RIG-I stimulation in CAR-T cells supports persistence, effector-memory differentiation, and reduced exhaustion. These findings suggest that targeting RIG-I, either through genetic modulation or RNA-based approaches, may provide a novel avenue to regulate T cell-based cancer therapies. Further research is needed to delineate the broader implications of RIG-I signaling in tumor-specific T cells, particularly in combination with existing immunotherapies.

**Table 3:** Summary of RIG-I influence on CD8+ T cells in antitumor immunity.

Modification	Functional Roles in CD8+ T cells	References
shRNA RIG-I knockdown	RIG-I acts as a checkpoint molecule in tumor-specific T cells Knockdown of RIG-I increased anti-apoptotic proteins (Bcl-2/Bcl-xL) and reduces caspase-3 mediated apoptosis RIG-I is a negative regulator of Akt, altering T cell metabolism RIG-I can sequester HSP90 and thus restrain STAT5 activation, inhibiting T cell cytotoxicity	(68) Duan et al. (2024) (69) Jiang et al. (2023)
CAR-T cell	RN7SL1 CAR-T cells maintained an effector-memory state, and exhibited enhanced antitumor efficacy and synergy using anti-CTLA-4 therapy	(70) Johnson et al (2021)

### Challenges and future directions for STING and RLR-based therapeutics

In summary, while both STING and RIG-I signaling pathways can modulate T cell responses, their therapeutic potential in cancer deserves further investigation. A key limitation of these pathways in T cells is the delicate balance between promoting effector function and driving apoptosis (50, 52, 55–60). High-intensity STING activation can induce T cell apoptosis, especially in high-affinity T cells, potentially undermining therapeutic efforts. However, when carefully tuned, STING can enhance the antitumor effects of low affinity tumor-specific T cells, potentially overcoming challenges associated with weakly immunogenic tumors (61). Moreover, recent studies have demonstrated the potential of leveraging STING signaling to maintain CD8+ T cell stemness (64, 65), offering promise for enhancing T cell persistence and function, especially in combination with other immune-modulatory therapies. Similarly, while RIG-I deficiency may improve CD8+ T cell effector functions (68, 69), other studies demonstrate that selective stimulation of RIG-I in CAR-T cells can improve durability and antitumor efficacy (70). These discrepancies highlight the need for continued investigation on how to best leverage nucleic acid receptor signaling to augment CD8+ T cell antitumor responses.

Moving forward, key areas of research may seek to better characterize STING and RIG-I signaling in CD8+ T cells. Further, emphasis should be on identifying the precise dosages and timing of agonist administration to prevent unwanted apoptosis while enhancing effector and memory responses. Investigating how other factors in nucleic acid sensing pathways can synergize with checkpoint blockade therapies and T cell-engineering approaches is another exciting frontier. Moreover, developing targeted methods to specifically manipulate T cell-intrinsic nucleic acid signaling may be critical in avoiding off-target effects and improving the therapeutic index. Finally, understanding how the tumor microenvironment modulates these pathways in T cells is essential to fine-tuning such therapies. The ongoing challenge lies in ensuring that these pathways can be harnessed to selectively enhance T cell function in a controlled manner to ultimately improve antitumor immunity.

## Natural killer receptors in CD8+ T cells

Natural killer receptors (NKRs) on T cells play a crucial role in bridging innate and adaptive immunity. While invariant natural killer T cells (NKT) are traditionally classified as innate immune cells due to their conserved TCR clonality, certain subsets of CD8+ T cells expressing NK markers maintain TCR diversity and are firmly embedded within the adaptive immune system (4, 71). Notably, human CD8+ T cells can express both inhibitory and activating NKRs, allowing for innate-like recognition of stressed, transformed, or infected cells (4, 71). Inhibitory NKRs, including killer-cell immunoglobulin-like receptors (KIRs) and NKG2A, recognize MHC ligands on healthy cells, acting as immune checkpoints to prevent autoreactivity (4). In contrast, activating NKRs, such as NKG2C, NKG2D, NKp44, and NKp30, bind stress-induced ligands found primarily on aberrant cells and promote cytotoxic responses, where NKG2D has been the most extensively studied to augment CD8+ T cell antitumor immunity (4). Together, NKRs can induce inhibitory or stimulatory responses in CD8+ T cells in different disease contexts. While also studied in gamma-delta T cells (72), we focus on NKRs in αβ CD8+ T cell antitumor immunity.

### NKG2A acts as a checkpoint receptor in CD8+ T cells

**Figure 3:**
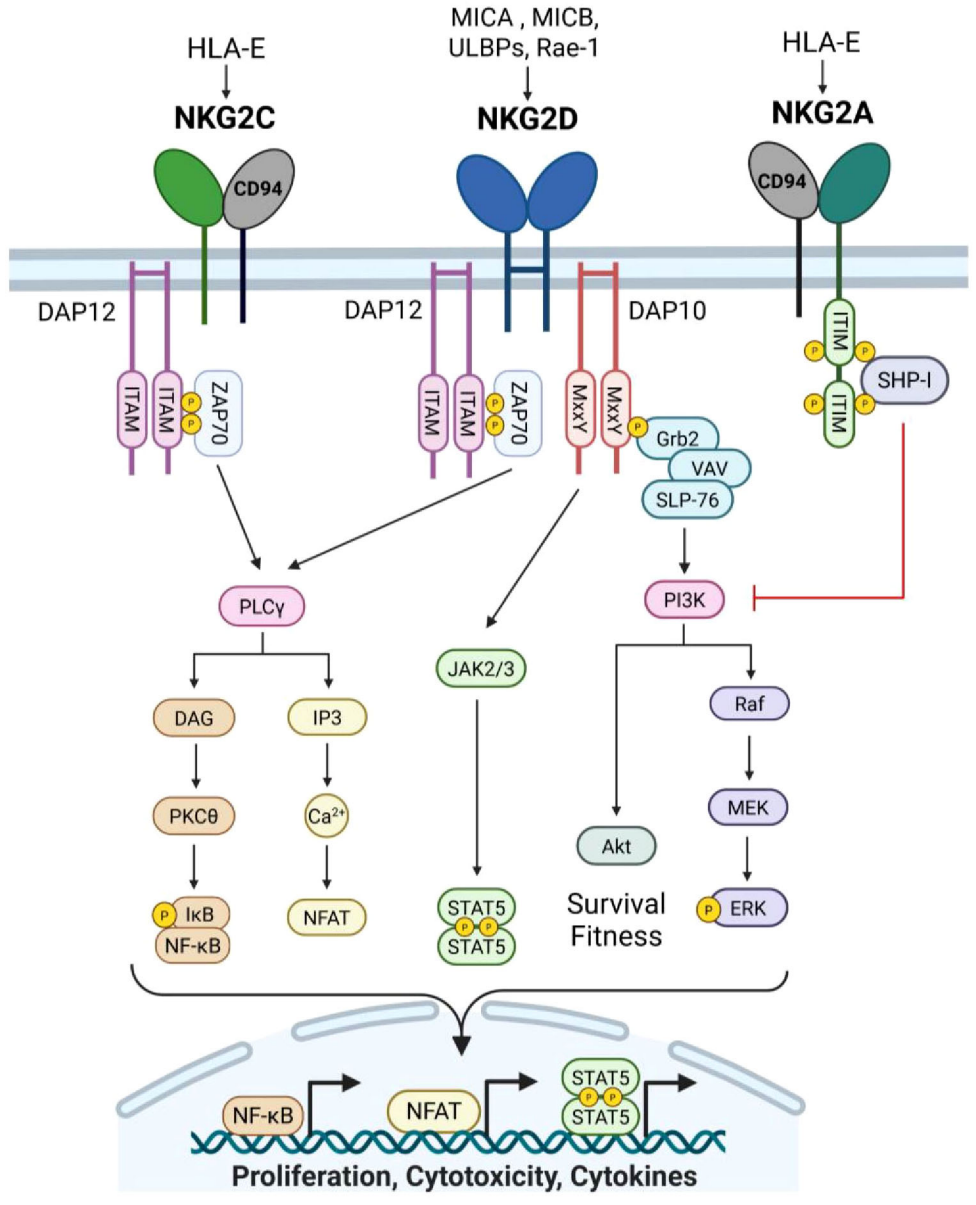
Natural killer receptor group 2 signaling. NKG2A and NKG2C form receptors with CD94 and recognize non-classical MHC-I molecule, HLA-E, bound to self-peptide. NKG2A is an inhibitory receptor that contains two ITIM motifs that are phosphorylated upon association with HLA-E/peptide complexes. SHP-I is recruited to the phospho-ITIMs to facilitate downstream inhibitory signaling of other activating NK receptors, particularly at the PI3K cascade. Conversely, NKG2C is an activating receptor and binds HLA-E/peptide complexes with lower affinity. Binding results in phosphorylation of ITAMs in the associated intracellular signaling molecule, DAP12, to propagate activating signals. DAP12 phosphorylation recruits ZAP-70 which transduces signal via PLCγ, leading to NF-kB activation as well as NFAT activity. Similarly, activating receptor NKG2D propagates signal through DAP12/ZAP-70, culminating in a cytotoxic immune response. Additionally, NGK2D association with DAP10 can promote cytokine production and survival programs. The MxxY motifs in DAP10 are phosphorylated upon binding of NKG2D to stress-induced ligands, which recruits Grb2, and forms a complex with VAV and SLP-76. Vav then promotes PI3K to drive Akt as well as phosphitylation of ERK, promoting cellular proliferation and survival. Lastly, DAP10 signaling can intersect with IL-15 receptor signaling via JAK2/3, resulting in STAT5 phosphorylation and transcriptional activity to drive cytokine expression. Created with BioRender.com.

NKG2A has emerged as a critical immune checkpoint receptor in CD8+ T antitumor immunity. NKG2A forms a heterodimer with CD94 and recognizes self-peptide bound to non-classical MHC-I molecule, HLA-E, which is widely expressed in different cancer types (73–75). Receptor engagement leads to intracellular phosphorylation of the ITIM domain to recruit SHP-I and suppress proximal TCR and activating NKR signaling (73–75) (**Figure 3**). Recent reports have indicated that NKG2A is induced by TCR engagement and upregulated in CD8+ TILs under chronic stimulation to suppress effector function similarly to PD-1, TIGIT, and TIM-3 (73–76). Interestingly, Montfoort et al. (74) leveraged NKG2A by demonstrating that administration of a peptide cancer vaccine in murine tumor models increased the expression on NKG2A on CD8+ TIL; however this elevated expression allowed for superior NKG2A blockade, thus improving therapeutic efficacy (74). Indeed, NKG2A-blockade (Monalizumab) has shown promising clinical efficacy, particularly in combination with anti-PD-1, by disrupting NKG2A-HLA-E interactions on cancer cells and reinvigorating exhausted TILs (77). While PD-1 is expressed upon T cell activation and maintained on responding cells, NKG2A appears to be upregulated at later timepoints, specifically on cytotoxic CD8+ T cells (76, 78). This phenomenon was illustrated by Borst et al. (78) where NKG2A expression on CD8+ tumor-specific T cells was increased following repeated antigen stimulation at later timepoints, and was maintained during cell division (78). This expression pattern was akin to late exhaustion markers, TIM-3 and CD39, while PD-1 and LAG-3 were upregulated within hours of stimulation with expression varying during proliferation (78). Given these findings, NKG2A blockade may hold promise in boosting antitumor efficacy after application of first line therapies or in other checkpoint non-responsive patients. Lastly, recent studies have revealed that NKG2A+ CD8+ T cells can exhibit transcriptional profiles distinct from other inhibitory KIR expressing CD8+ subsets, suggesting an influence of NKRs on CD8+ T cell fate (4, 71, 79). In 2023, Choi et al. demonstrated that human KIR+ CD8+ T cells were terminally differentiated with evidence of senescence and poor proliferative capacity, while NKG2A+ CD8+ T cells maintained a cytotoxic effector state (71). As signaling through classical T cell inhibitory receptors can alter downstream transcriptional and epigenetic states, whether NKG2A influences similar pathways warrants future investigation to better characterize the extent that this innate inhibitory molecule influences CD8+ T cell fate, function, and survival in the TME.

### NKG2C promotes CD8+ T cell cytotoxicity

In contrast to NKG2A, NKG2C functions as an activator by binding to HLA-E and signaling through the immune-tyrosine activating motif (ITAM)-containing adaptor, DAP12 (DNAX-activating protein 12) (**Figure 3**). NKG2C ligation alone can activate CD8+ T cells, bypassing TCR signaling and promoting cytotoxicity against HLA-E-expressing targets (4, 80). This suggests that NKG2C signaling may serve as an alternative activation pathway in CD8+ T cells, independent of classical TCR engagement. More research is needed to delineate differences in CD8+ T cell responses under TCR versus NKG2C activation, and if stimulation through NKG2C can allow CD8+ to be more resistant to exhaustion. NKG2C has also been suggested to alter CD8+ T cell differentiation. Namely, downregulation of BCL11B in CD8+ T cells has been linked NKG2C expression. BCL11B is a transcription factor essential for maintaining T cell identity, which suggests NKG2C may shift CD8+ T cells towards an NK-like phenotype (81, 82). In 2024, Lupo et al. demonstrated that using NKG2C+ CD8+ T cells as the source for engineered T cell therapies enhanced antitumor efficacy (83). Specifically, CD19 CAR-T cells generated from NKG2C+ CD8+ T cells exhibited superior tumor killing, persistence, and significantly reduced cytokine release syndrome compared to conventional CAR-T cells (83). Given these promising results, future studies may seek to leverage NKG2C to modulate CD8+ T cells for adoptive cell therapy.

### NKG2D can influence CD8+ T cell differentiation and cytotoxicity

Natural killer group 2 member D (NKG2D) is a type II transmembrane activating receptor that is expressed on NK cells, naïve human, and activated cytotoxic CD8+ T cells (4, 84) (**Figure 3**). As a member of the killer cell lectin-like receptor subfamily K, NKG2D (*Klrk1*) engages an array of MHC class I-like ligands that are upregulated in response to cellular stress, enabling promiscuous recognition of infected or transformed cells (4, 84, 85). In humans, NKG2D recognizes MICA, MICB, and six members of the UL16-binding protein (ULBP) family, whereas in mice, its ligands include isoforms of the Rae1 family, MULT1, and H60 (4, 84, 85). Structurally, NKG2D forms a homodimer and contains a short cytoplasmic tail that lacks intrinsic signaling domains (4, 84, 86). As such, NKG2D relies on adaptor DNAX-activating proteins, DAP10 and/or DAP12, to transduce intracellular signals (4, 84). Two isoforms of NKG2D exist, differing in length and activating distinct signaling pathways. The long NKG2D-L isoform utilizes DAP12, which contains an ITAM motif that phosphorylates Src-family kinases SYK and ZAP70, enhancing cytotoxicity and effector function (4, 84–86). Notably, more than twenty DAP12-associated receptors, including TREM1/2 and KIRs, have now been identified, highlighting the expanding landscape of NK-like signaling pathways in adaptive immunity (4, 84). Conversely, the short NKG2D-S isoform signals through DAP10, which contains the YxxM motif, recruiting GRB2 and the p85 subunit of PI3K to activate downstream VAV and PI3K signaling cascades (4, 84–87). In addition to signaling distinctions, while mice express both DAP10 and DAP12, human CD8+ T cells rely exclusively on DAP10 (4, 84, 87). However, engineering efforts have demonstrated that fusing NKG2D with DAP12 in human CD8+T cells conferred enhanced effector function, underscoring the adaptability of NKR signaling in T cells.

Similarly to DAP10, the costimulatory molecule CD28 also contains the YxxM motif, leading to early reports investigating the distinction between CD28 and NKG2D/DAP10 co-stimulation in CD8+ T cells (88–90). Key differences between timing of stimulation and signaling outcomes pointed to CD28 being primarily involved in T cell priming while NKG2D provides additional stimulatory signals to already activated, responding cells (84, 88, 89). For instance, unlike CD28 ligands, which are largely restricted to APCs, NKG2D ligands are broadly expressed, as their upregulation is driven by cellular stress in various tissues. Moreover, several studies have indicated that NKG2D differentially regulates CD8+ T cell fate compared to co-stimulation through CD28 (84, 88, 89). NKG2D weakly activates mTORC1 signaling, leading to transcriptional upregulation of memory-associated genes such as *Sell* and *Il7r* in CD8+T cells, whereas CD28 strongly activates mTOR, driving terminal effector differentiation through activity of T-bet, Blimp-1, and IRF4 transcription factors (87, 89). NKG2D has also been shown to enhance IL-15-mediated PI3K activity in activated CD8+ T cells, reinforcing memory fate commitment and survival (84, 87, 91). Notably, CD8+ T cells lacking NKG2D exhibited reduced memory potential following lymphocytic choriomeningitis virus (LCMV) infection due to impaired PI3K activation, limiting pro-survival factors necessary for long-term persistence (84). In cancer models, blockade of NKG2D on effector CD8+ T cells similarly impaired memory formation (91). Lastly, in the absence of traditional co-stimulation, NKG2D has been shown to play a compensatory role in CD8+ T cell differentiation. For example, Harris et al. (92) demonstrated that loss of TCF1 in mature CD8+ T cells led to reduced CD28 expression and a compensatory upregulation of NKG2D (92). Further, blocking NKG2D signaling in TCF1-/- CD8+ T cells impaired cytolytic function (92), suggesting that NKG2D was essential to providing co-stimulatory signals to maintain CD8+ T cell antitumor responses. Notably, similarly to CD28, chronic NKG2D activation has been linked to diminished T cell responses in the TME (87), suggesting a complex balance between productive co-stimulation and terminal effector differentiation and exhaustion in antitumor immunity.

In 2023, Lerner et al. demonstrated that NKG2D stimulation alone primarily mediated immune activation (93). OT-I NKG2D+ T cells were cultured with MHC-I knockout CT2A cells (CT2A-TRP2-βmKO) with or without NKG2D blockade and OVA-expressing APCs. T cells failed to clear MHC-I null cancer cells upon NKG2D-blockade and, in the absence of blockade, T cells only eliminated CT2A-TRP2-βmKO cells in the presence of OVA-expressing APCs in a Fas/FasL-dependent manner (93). Together, these experiments revealed that NKG2D-dependent cytotoxicity requires prior TCR engagement. Moreover, this study illustrated the benefits of NKG2D stimulation in the context of MHC-I loss, a common immune evasion strategy employed by tumors. Previous studies have indicated that checkpoint blockade-enhanced tumor killing persisted even in MHC-I-deficient tumors, and this effect was later attributed to NKG2D+ CD8+ T cells rather than NK cells (93, 94). Lerner et al. used MHC-I deficient tumor models to isolate the role of NKG2D through stress-ligand recognition, rather than MHC-I for cytotoxicity (93). These findings highlight the potential of NKG2D-targeted strategies to enhance CD8+ T cell responses against MHC-I-deficient tumors. To this point, as NKG2D activating ligands are not expressed on healthy cells, this may also reduce instances of graft-versus-host disease (GVHD) following administration of adoptive cell therapies, thus maintaining potent antitumor activity while limiting off-target toxicity (92). Together, these findings support further exploration of modulating NKG2D as an exciting avenue for improving CD8+ T cell-based immunotherapy.

### NKG2D-based CAR-T cell therapies: advancements in design and safety

Several studies have reported on NKG2D-based CAR-T cells in targeting different cancers (85, 95–98). Celyad Oncology has been at the forefront of developing these NKG2D CAR-T cells (95, 99). The original construct featured a full-length human NKG2D receptor fused to CD3ζ (NKG2D-ζ), while subsequent iterations incorporated additional costimulatory domains, such as 4-1BB (NKG2D-BBζ), to enhance persistence and effector function (98, 100–102). In 2019, Yang et al. identified that glioblastomas express high levels of NKG2D ligands, prompting an investigation into the therapeutic potential of NKG2D-BBζ CAR T cells (103). In xenograft U-251MG models, NKG2D-BBζ CAR T cells exhibited superior tumor infiltration and achieved complete tumor clearance within 21 days compared to CD19-BBζ CAR T cells (103). Importantly, safety evaluations revealed no signs of treatment-associated toxicities, abnormal proliferation, or genomic instability (103). These promising findings extended beyond glioblastoma, as NKG2D-ζ CAR T cells also demonstrated efficacy in acute myeloid leukemia (AML) and T cell acute lymphoblastic leukemia (T-ALL) (101), reinforcing the potential of NKG2D-directed CAR therapies in cancer immunotherapy.

Given the advantages of NKG2D-mediated signaling in regulating CD8+ T cell function, recent research has sought to optimize CAR designs by incorporating NKG2D signaling adapters. In 2021, Ng et al. engineered a novel NKG2D-based CAR by fusing the ectodomain of NKG2D to 4-1BB and DAP12 (104). Functional assessments demonstrated that while both the DAP12-containing CAR and its predecessors exhibited comparable tumor-killing, the DAP12 construct produced lower levels of IFNγ, TNFα, and IL-2, during tumor cell lysis (104). Given that excessive cytokine secretion is a primary driver of CAR-associated toxicities like cytokine release syndrome (CRS), these findings suggest that NKG2D-DAP12 may offer a more tolerable CAR-T cell design. The efficacy and safety profile was validated in a colorectal cancer (HCT116) xenograft model, where both CD3ζ and 4-1BB/CD3ζ CAR-T cells effectively reduced tumor burden, but only the DAP12 construct achieved complete eradication (104). Phenotypic analysis revealed that NKG2D-DAP12 CAR-T cells preferentially differentiated into effector memory cells (CD45RO+CCR7+), a characteristic linked to improved persistence and long-term therapeutic efficacy (104). Moreover, while both the DAP12- and CD3ζ-CARs eliminated tumors, mice treated with CD3ζ-based CARs exhibited significant weight loss (>15%), signs of graft-versus-host disease (GvHD), and hepatic necrosis and bile duct hyperplasia. In contrast, mice treated with DAP12 CAR-T cells remained tumor-free up to 150 days with no evidence of immune-related toxicities (104). Additionally, even at doses exceeding the human therapeutic range, DAP12-based CAR-T cells did not induce significant adverse effects on body weight, temperature, or organ function with similar tumor eradication and 100% survival in a SKOV3 ovarian cancer xenograft model (104).

Recent studies have investigated the ability of NKG2D CAR-T cells to target and eliminate senescent cells. Cellular senescence contributes to age-related tissue decline and is marked by permanent cell cycle arrest and the senescence-associated secretory phenotype (SASP) (105, 106). Aging cells can upregulate NKG2D ligands as markers of cellular stress, offering a promising target for treating aging-related diseases (105). Indeed, NKG2D-BBζ CAR-T cells effectively diminished senescent human cells, alleviating aging-associated pathologies and improving physical performance in mice (105). Further, NKG2D-BBζ CAR T-cells were able to eliminate naturally occurring senescent cells in aged nonhuman primates, without adverse effects (105). Similarly, in 2024, Deng et al. explored the therapeutic potential of NKG2D-ζ CAR T cells in targeting senescent cells in the brain (106). Using mouse embryonic fibroblasts (MEFs) and astrocytes (AST) as models for cellular senescence, NKG2D-ζ CAR T cells demonstrated strong cytotoxic activity, with minimal impact on non-senescent cells (106). This study provides the first evidence that NKG2D-CAR T cells can specifically target senescent brain cells, offering a novel strategy for addressing senescence-related brain disorders (106). Together, these studies offer a novel strategy for combating age-associated pathologies beyond cancer by leveraging NKG2D.

An emerging area of research extending these concepts further involves the development of γδ T-cell adoptive therapies. Because they do not rely on classical MHC-mediated antigen presentation, they offer significant potential as an allogeneic, off-the-shelf cell therapy product. Recent strategies are exploring the use of NKRs to augment the antitumor efficacy of γδ T-cells. Specifically, Vδ1 T-cells, a subset of γδ T-cells, have demonstrated remarkable antitumor cytotoxicity driven by elevated NKR expression (107). Like conventional CAR T-cell therapies, γδ T-cells can be genetically modified to express NKG2D-ligand recognizing CARs. A key advantage of NKG2D CAR γδ T-cells over traditional αβ T-cells is their reduced susceptibility to activation induced cell death (108), potentially translating into enhanced persistence and therapeutic efficacy.

In summary, NKG2D-based CAR T cell therapies have demonstrated significant potential in targeting various malignancies, with iterative improvements enhancing their efficacy and safety profiles. The development of the second-generation NKG2D-DAP12 CAR represents a crucial advancement. These findings underscore the promise of NKG2D-DAP12 CAR T cells as a next-generation immunotherapy strategy, warranting further clinical investigation to translate these benefits into effective treatments for cancer patients.

### Leveraging DNAX-activating proteins to enhance CAR-T cell persistence and limit toxicity

Beyond the full NKG2D CAR T cell design, several reports have leveraged its intracellular signaling components, DAP10/DAP12, to a range of antigen-targeting domains (109–114). In 2021, Sun et al. investigated the efficacy of CD19 targeting DAP12-BB CAR-T cells compared to conventional CAR constructs in preclinical models of B-cell acute lymphoblastic leukemia (NALM6) and pancreatic cancer (AsPC-1) xenografts (110). While tumor control was comparable, DAP12-BB CAR T cells displayed enhanced proliferation and persistence in peripheral blood (110). While the authors do not mechanistically investigate this finding, later works suggest this enhanced fitness may be a result of NF-κB and/or PI3K-Akt-mTOR activity in DAP12 CAR T cells. Encouraged by these findings, a Phase 1 clinical trial (ChiCTR1800016584) was initiated for patients with relapsed/refractory (r/r) B-ALL (110). Following lymphodepleting chemotherapy, patients received either CD19-BBζ or DAP12-BB CAR-T cells (110). Cytokine profiling revealed patients receiving DAP12-BB CAR therapy had lower circulating IL-6 and IL-10 levels, with only one instance of grade 3 CRS. In contrast, all patients in the control group experienced grade 2 or 3 CRS (110). Additionally, DAP12-BB CAR-T cells expanded more rapidly, peaking at day seven compared to day fourteen in the CD19-BBζ CAR group (110). All patients in the trial ultimately achieved complete response (CR); however, relapse rates differed significantly—three out of four control patients relapsed within five months, with two succumbing to the disease shortly thereafter. Only two patients from the DAP12-BB CAR group experienced relapse disease, occurring at nine and fourteen months, and all patients survived by the final follow-up (110). These findings suggest that CD19-DAP12-BB CAR T cells may offer improved persistence, faster tumor clearance, and reduced toxicity, making them a promising alternative to conventional second-generation CAR T therapies for r/r B-ALL.

Yielding auxiliary results, Li et al. (114) engineered a dual-antigen targeting system using native NKG2D on T cells to engage NKG2D ligands on tumor cells, while simultaneously incorporating an anti-glypican 3 (GPC3) scFv for additional specificity (114). Here, the recombinant GPC3-DAP10-BBζ construct expressed the full-length DAP10 fused to 4-1BB and CD3ζ activation domains (114). In a heterogeneous xenograft model, GPC3+tdTomato+ and GPC3-EGFP+ SK-Hep1 cells were mixed and transplanted into NSI mice, creating a diverse TME to assess the targeting capacity of GPC3-DAP10-CAR T cells in a more clinically relevant solid tumor system (114). Indeed, the tandem GPC3-DAP10-CAR T cells exhibited dual-antigen-targeting capacity, suppressing the growth of heterogeneous tumors *in vivo* (114). Thus, this novel dual-targeting system extended the recognition profile of CAR-T cells toward tumors with more heterogenous antigen expression, providing an innovative tumor targeting strategy.

In addition to improved tolerability and sustained tumor control, DAP CAR-T cells were studied to address limitations of tonic signaling in second-generation CAR constructs. Tonic CAR signaling, which hinders therapeutic efficacy, results from sustained, uncoordinated CD3ζ activation, leading to poor proliferation and accelerated exhaustion. DAP12, unlike CD3ζ, contains ITAM and ITIM domains, which regulate signaling strength through recruitment of SHP-1, resulting in suppression or promotion of tonic signaling (109, 111). Zheng et al. (111) demonstrated that a Lym-1 targeting DAP10/12 CAR reduced expression of T cell exhaustion markers, PD-1 and LAG-3 during response to B-cell lymphoma (111). While, second-generation CARs failed to clear Raji tumors, the DAP10/12 CAR-T cells significantly extended survival and elicited durable tumor control, even in rechallenge settings (111). This study also addressed another common challenge in CAR therapies, where treatment with antigen-targeting CAR-T cells results in downregulation of that antigen on tumor cells. *In vitro*, second-generation CARs led to significant CD19 downregulation and modest Lym-1 reduction, while DAP10/12 CARs did not induce antigen loss (111). Together, these findings suggest that DAP10/12-based CAR-T cells could overcome expansion and exhaustion challenges while enhancing antitumor responses in B-cell lymphoma while curbing immune evasion by antigen loss.

With the promising results from DAP-based CAR designs, Xu et al. (112) then evaluated the therapeutic potential of CAR-T cells targeting PTK7 on ovarian cancer cells through TREM1/DAP12 signaling (112). TREM1 is a DAP12-associated receptor known to amplify immune activating signals (112). Here, CAR-T cells incorporating TREM1/DAP12 signaling exhibited strong cytotoxic activity against PTK7-cancer cells, eradicating tumors *in vivo* (112). Similarly, Nie et al. (113) developed a novel DLL3-targeted CAR T cell therapy incorporating TREM1/DAP12 (DLL3-DT) for the treatment of small cell lung cancer (SCLC) (113). Compared to a second-generation DLL3 CAR, DLL3-DT CAR-T cells eliminated SCLC xenograft tumors and mice remained tumor-free through the study endpoint without signs of toxicity, and prevented tumor recurrence prevented tumor recurrence following secondary challenge (113). Testing in a patient-derived xenograft (PDX) SCLC model confirmed complete tumor regression with no relapse by seventy days (113). Gene expression analysis showed that both control and DLL3-DT CAR T cells exhibited similar activation profiles, however, DLL3-DT cells were enriched for pathways associated with memory and NK-mediated cytotoxicity, suggesting that DT CARs may mimic myeloid or NK cell activation pathways (113). These findings support DLL3-DT CAR-T cells as a promising therapeutic strategy for SCLC, offering enhanced durability, tumor clearance, and long-term immune surveillance.

By fine-tuning the balance between replicative capacity and memory cell persistence, DAP-based CARs may offer superior durability and therapeutic longevity while mitigating the excessive cytokine release and immune-related toxicities commonly associated with traditional CAR constructs. These advancements highlight the potential of innate-like signaling molecules (DAP10/12 and TREM1) in CAR T cell therapies as a safer and more effective strategy for both solid and hematologic malignancies (summarized in **Table 4**).

**Table 4:** Summary of NKR influence on CD8+ T cells in antitumor immunity and NKR-based CAR-T cell efficacy.

NK Receptor	Functional Roles in CD8+ T cells	References
NKG2A	NKG2A acts as a checkpoint molecule with similar kinetics to TIM-3 Blockade with Monalizumab enhanced CD8+ T cell cytotoxicity and synergized with other checkpoint blockade therapies NKG2A expressing CD8+ T cells exhibit more robust effector responses	(71) Choi et al. (2023) (73) Borst et al. (2021) (74) Monfoort et al. (2018) (77) Andre et al. (2018) (78) Borst et al. (2022)
NKG2C	NKG2C can bypass TCR activation to induce CD8+ T cell cytotoxicity NKG2C+ CAR-T cells exhibit superior persistence, cytotoxicity, and tolerability	(80) Kho et al. (2022) (83) Lupo et al. (2024)
NKG2D	NKG2D supports memory differentiation in CD8+ T cells NKG2D/Dap10 weakly activates mTORC1 signaling leading to upregulation of memory genes, *Sell* and *Il7r* Enhances IL-15-mediated PI3K activity to reinforce CD8+ T cell memory differentiation NKG2D-deficient CD8+ T cells exhibit impaired survival and persistence, and blockade interferes with memory development TCF1 can regulate NKG2D expression on tumor-specific CD8+ T cells	(84) Wensveen et al. (2018) (87) Prajapati et al. (2018) (88) Rajasekaran et al. (2010) (91) Perez et al. (2019) (92) Harris et al. (2023)
NKG2D	NKG2D can compensate for loss of CD28 signaling to boost cytotoxicity via granzyme B production. NKG2D-mediated cytotoxicity requires prior TCR engagement NKG2D+ CD8+ T cells direct anti-tumor responses to MHC-I deficient tumors	(90) Markiewicz et al. (2016) (92) Harris et al. (2023) (93) Lerner et al. (2023)
CAR-T cell Design	Functional outcomes of NKG2D and DAP10/12 modified CAR-T cells	References
NKG2D-ζ NKG2D-BBζ	Upregulation of NKG2D on senescent cells was targetable with NKG2D CAR-T cells which induced cytotoxic responses to eliminate senescent cells in age-associated diseases NKG2D-CAR-T cells exhibit superior tumor infiltration, persistence, and cytotoxicity compared to CD19-CAR-T cells Greater tolerability and lower treatment-associated toxicities	(95) Michaux et al. (2022) (99) Lonez et al. (2018) (100) Obajdin et al. (2024) (101) Driouk et al. (2020) (103) Yang et al. (2019) (105) Yang et al. (2023) (106) Deng et al. (2024)
NKG2D-BB-DAP12	Lower systemic levels of IFN-γ, TNF-α, and IL2 during anti-tumor response resulted in significantly fewer instances/grade of CRS	(104) Ng et al. (2021)
DAP12-BB DAP12-BBζ DAP10-BBζ	Enhanced CAR-T cell persistence, proliferation, and fitness through NF-kB and/or PI3K-Akt-mTOR activity Lower serum cytokine levels (IL-6, IL-10) and reduced instances of > grade 2 CRS Increased speed of tumor clearance and greater long-term tumor control	(109) Yu et al. (2024) (110) Sun et al. (2021) (114) Li et al. (2022)
DAP10/DAP12	Reduced tonic signaling in CAR-T cells Less exhausted as evidence by reduced PD-1 and LAG-3 expression on CAR-T cells Superior and more durable tumor control upon secondary tumor challenges DAP10/DAP12 CAR-T cells did not induce antigen escape mechanisms in murine tumor models	(111) Zheng et al. (2020)
TREM1/DAP12	Robust cytotoxic effects and long-term tumor control Modified CAR-T cell gene expression to enrich for pathways associated with memory and NK-mediated cytotoxicity	(112) Xu et al. (2023) (113) Nie et al (2024)

### Challenges and Future Directions for NKR-based therapeutics

Stimulating NKR signals on CD8+ T cells represent a critical intersection between innate and adaptive immunity, influencing differentiation, effector function, and therapeutic potential in immune-mediated diseases, particularly cancer. While inhibitory NKRs, such as NKG2A and KIRs, serve as immune checkpoints to regulate CD8+ T cell activity, their blockade has emerged as a promising strategy to reinvigorate exhausted TILs (71, 73, 74, 77, 78). Conversely, activating NKRs, including NKG2D and NKG2C, enhance CD8+ T cell cytotoxicity, offering alternative mechanisms for targeting stressed or transformed cells, particularly in the context of MHC-I downregulation (93). NKG2D has gained significant attention for its role in CD8+ T cell differentiation and cytotoxicity. The ability of NKG2D signaling to complement traditional co-stimulatory pathways and drive memory formation underscores its importance in adaptive immunity (84). Furthermore, its application in immunotherapy, particularly through CAR engineering, has yielded promising results – NKG2D-based CAR T cell therapies have demonstrated potent antitumor activity while mitigating adverse events, particularly with the incorporation of DAP10 and DAP12 signaling components (104, 109–113). Recent advances in NKG2D-CAR T cell therapies extend beyond oncology, with emerging applications in targeting senescent cells implicated in age-related diseases. Studies leveraging NKG2D-CAR constructs to selectively eliminate senescent cells in aged models highlight its potential to modulate immune responses beyond cancer (105, 106), opening avenues for treating degenerative disorders.

Despite these promising developments, several challenges remain. The heterogeneity of tumor microenvironments, immune evasion mechanisms, and potential off-target effects pose barriers to widespread clinical implementation. Additionally, long-term persistence and functionality of engineered T cells need to be optimized to prevent exhaustion and loss of efficacy. Future research should focus on DAP/TREM signaling dynamics to refine CAR-T cell persistence while maintaining low toxicity profiles and enhanced antitumor responses. Specifically, how NKR signals influence CD8+ T cell stemness and exhaustion have not been explored. Efforts to better understand the interplay between NKRs and other stimulatory or inhibitory pathways will also be crucial for designing next-generation therapies.

These findings underscore the dynamic role of NKRs in shaping CD8^+^ T cell function and highlight the translational potential of NKG2D-based strategies in immunotherapy. With ongoing clinical advancements, the integration of NKG2D and its signaling adaptors into adoptive T cell therapies holds promise for improving patient outcomes in cancer and beyond, reinforcing the potential of harnessing innate-like signaling pathways in adaptive immune responses.

## Conclusion

**Figure 4:**
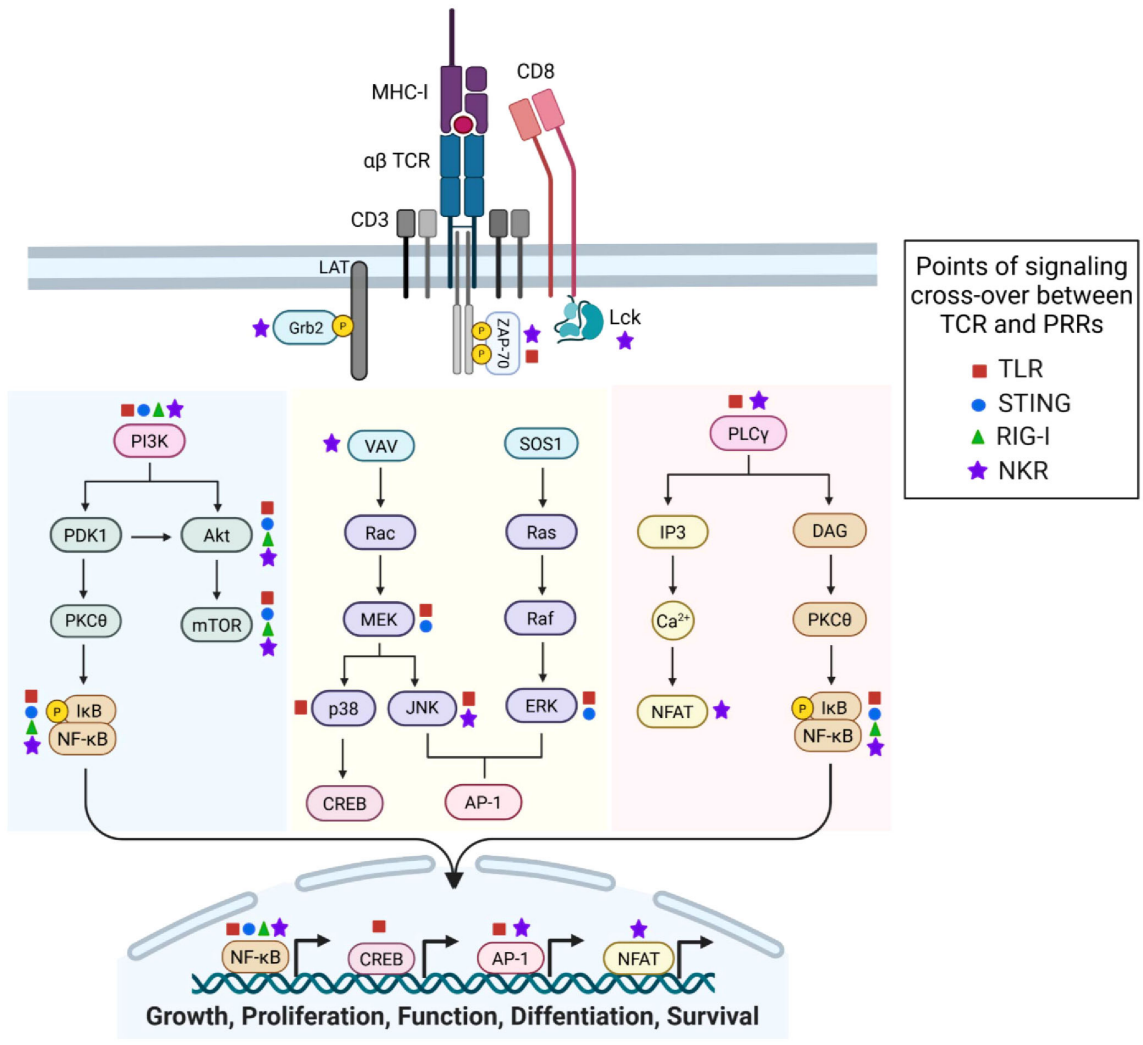
Cross-over between innate-immune receptor and T cell receptor (TCR) signaling – opportunities for synergy in immunotherapy. The CD8 co-receptor and αβ TCR associate with MHC-I/peptide complexes. Peptide binding activates Src-family kinase, Lck, which phosphorylates the ITAMs in the TCR CD3ζ signaling domain. Phosphorylation recruits ZAP-70 and phosphorylation of scaffold protein LAT to propagate the TCR signal. PI3K signaling (shaded blue) leads to PDK1 induction of PKCθ and subsequent activation of NF-kB. PI3K can also activate Akt to induce mTOR-mediated control of T cell fitness and metabolism. Phosphorylated Grb2 can complex we molecules such as VAV and SOS1 to induce MAPK cascades (shaded yellow) via RAC-MEK-JNK/p38 or Ras/Raf/ERK, culminating in activation of CREB and/or AP-1 transcription factors. Lastly, phosphorylation of LAT leads to induction of PLCγ-mediated signaling (shaded pink). PLCγ regulates (1) calcium flux (Ca^2+^) and NFAT activity, and (2) DAG activation of PKCθ and thus, NF-kB. Together, TCR signaling results in NF-kB, CREB, AP-1, and NFAT transcriptional activity to drive cell growth, proliferation, function, differentiation, and survival. Signaling molecules documented to be influenced by innate-receptor signaling are indicated by colored symbols (TLR, red square. STING, blue circle. RIG-I, green triangle. NK-receptors, purple star). Created with BioRender.com.

The interplay between innate immune sensors—TLRs, STING, RLRs, and NKRs—in CD8+ T cells is a growing area of research with major implications for adaptive immunity and cancer immunotherapy. Traditionally, studied in innate immune cells, these sensors significantly influence CD8+ T cell function and fitness and exhibit synergistic overlap with TCR signaling pathways (**Figure 4**). Thus, integrating innate-like signaling into adaptive immune cells is a powerful strategy for enhancing immunotherapy and offers new therapeutic avenues. Targeting TLR, STING/RLR, and NKR pathways in CD8+ T cells hold promise for treating cancer and other immune-related conditions. Continued research is essential to overcome current challenges and translate these insights into effective clinical therapies.
